# Abdominal Stiffness Evaluation in Massage for Constipation

**DOI:** 10.3390/s21041192

**Published:** 2021-02-08

**Authors:** Yunyi Wang, Chiaki Sakakibara, Miho Shogenji, Mikako Yoshida, Tetsuyou Watanabe

**Affiliations:** 1 Graduated School of Natural Science and Technology, Kanazawa University, Kakuma-machi, Kanazawa 920-1192, Japan; wyy24678@gmail.com; 2 Home-Visit Nursing Care Station Yayanoie, 88 Suehiro, Komatsu 923-0954, Japan; chiaki@sorabuta.com; 3 Faculty of Health Sciences, Institute of Medical, Pharmaceutical and Health Sciences, Kanazawa University, 5-11-80 Kodatsuno, Kanazawa 920-0942, Japan; shogen@mhs.mp.kanazawa-u.ac.jp; 4 Department of Women’s Health Nursing & Midwifery, Tohoku University Graduate School of Medicine, Sendai 980-8575, Japan; mikako.yoshida.e2@tohoku.ac.jp; 5 Faculty of Frontier Engineering, Institute of Science and Engineering, Kanazawa University, Kakuma-machi, Kanazawa 920-1192, Japan

**Keywords:** abdominal stiffness, abdominal massage, stiffness sensor

## Abstract

According to the experience of nurses and physiotherapists, the abdomen of constipated people becomes softer after abdominal massage. However, the relationship between the decrease in abdominal stiffness and the benefits of abdominal massage has not been examined quantitatively and is unclear. Furthermore, devices for measuring stiffness have been designed to measure relatively hard areas such as the shoulders and do not take into account the lateral outflow of the target tissue, which can be a problem when measuring the stiffness of soft areas such as the abdomen. To address these issues, this study presents a stiffness sensor suitable for measuring abdominal stiffness and investigates the relationship between the reduction in abdominal stiffness and the benefits of abdominal massage. The solution to prevent the lateral outflow of the target is the realization of a stopper, including a contact detection device, which enables a wide-area contact around the targeted area. The sensor consists of a stopper, probe, spring, and time-of-flight (ToF) sensors. The probe and spring provide appropriate pressure and deformation to the abdomen, whereas the stopper prevents the probe from being pushed into the abdomen more than necessary. The ToF sensor measures the deformation length when the deformation is stopped by the stopper. The abdominal stiffness can be derived from the deformation length. The investigation results indicate that the reduction in abdominal stiffness corresponds to the improvement of the stool condition or the maintenance of a healthy stool condition, whereas the maintenance of abdominal stiffness indicates the maintenance or deterioration of the stool condition.

## 1. Introduction

The age of the Japanese population has been increasing year by year since 1950, and the care of older adults has become increasingly serious in recent years. By the end of 2016, the total population of Japan reached 122.693 million, of which 27.3% were over 65 years old [1]. Therefore, people from all circles seek solutions to problems related to the care of older adults. Among these, chronic constipation with regard to defecation is a major issue that cannot be ignored, as it always plagues older adults. This trend is observed in Japan as well as globally, with a prevalence of 30–70% [2,3,4].

Constipation is defined as a state in which feces cannot be defecated in sufficient quantity and with comfort [5]. It can be a complication of many diseases, such as diabetes and neurological diseases. However, according to surveys, in the vast majority of constipated older adults, no obvious pathological lesions can be found [6]. This means that as people age, nonpathological constipation symptoms become more common. Therefore, there is an urgent need to resolve constipation without damaging the bodies of older adults.

Popular chronic constipation in older adults is characterized by difficulties in defecation and slow-transit constipation. Digital disimpaction (the use of fingers to aid in the removal of stool from the rectum [7]) is a major issue in defecation difficulties. On the other hand, several methods have been utilized to solve slow-transit constipation in older adults, such as using laxatives. However, inappropriate use of laxatives causes diarrhea, which can cause further damage to the internal environment of the colon, worsening the symptoms of slow-transit constipation, and causing other harmful factors. One of the most appropriate methods for slow-transit constipation is abdominal massage, which has been proven to be clinically effective in several experiments [8,9,10,11,12,13].

Studies have shown that specific massage improves the temperature of the fingertip, has some positive effects on heart rate and blood pressure [14], stimulates skin blood flow [15], relieves stress and fatigue, and improves skin temperature [15,16]. Çevik et al. conducted a survey [17], showing that the most demanding issue in older adults is constipation caused by tension. Moreover, the study used a clockwise circular massage method that lasted for 30 days, and each massage lasted for 45–60 min to observe defecation after a period of massage, demonstrating the remarkable effect of abdominal massage on the treatment of constipation.

Regarding the effectiveness of abdominal massage, according to the experience of nurses and physiotherapists, the abdomen of people suffering from slow-transit constipation is often stiffer than that of people without constipation, whereas the abdominal feel of the constipated people softens after receiving abdominal massage. Tension in the rectus and transversus abdominis muscles results in poor bowel movement. This is considered to be reflected in abdominal stiffness. Massaging is considered to improve blood circulation, which in turn improves bowel movement and softening. Unfortunately, this relationship is based on interviews with nurses and has thus far been unclear. Accordingly, our purpose was to focus on abdominal stiffness or softness and explore the relationship between the changes in abdominal stiffness and the effectiveness (i.e., relief of constipation) of abdominal massage. The effect of massage is reflected in the fecal properties, but it takes several days to confirm it. If the change in abdominal stiffness can be correlated with the effect of massage, then the method and duration of the massage can be changed appropriately during massage. To address this, this study aims to design a stiffness sensor for the abdomen that is suitable for this research to measure and record abdominal stiffness. Additionally, this study aimed to investigate the defecation status of participants before and after abdominal massage and analyze the data to determine the relationship between the effectiveness of abdominal massage and reductions in abdominal stiffness due to massage.

Thus far, abdominal stiffness has not been considered an evaluation target. In contrast, stiffness of the neck and shoulder was evaluated using a muscle hardness tester, which is commercially available [18]. Unfortunately, this type of sensor cannot be used to evaluate abdominal stiffness because the abdominal area is softer than the stiff neck and shoulder, especially in older adults. One of the main issues in measuring the stiffness of soft abdominal regions is that when the tester is pushed into the abdomen, fat, and other abdominal tissues flow to the side, and the target tissue cannot be evaluated. One solution is to push the target tissue while making contact with a wide surface, which prevents the tissue from flowing to the sides. The probe for the tester should also be designed according to the softness of the abdomen. 

Sul et al. proposed a new stiffness measurement system that utilizes the lateral deformation profile of an object under indentation [19]. They defined stiffness k as the force acting on an object divided by the deformation. They utilized several force sensors for force measurement and contact detection and calculated the deformation indirectly according to Sneddon’s formula. The unique feature of their system is that users can choose a desired sensing range for the force measurement module, which helps to avoid measurement saturation. However, their system used force sensors in contact detection and minimized the threshold force of the force sensor by the lever mechanism to increase the sensitivity of the sensor so that the system could detect weak contacts. The accuracy of contact detection based on force sensors is not high, and the transmission efficiency (friction, deadweight, etc.) of the lever will further reduce its detection reliability. Moreover, their system can only measure substances with relatively uniform mechanical properties, which means that it may not be possible to measure the fat, muscles, and other tissues deep in the abdomen with reliable wide contact, preventing the tissues from flowing to the sides. Hence, it is not suitable for stiffness measurements in our abdominal massage experiment.

Sim et al. developed a portable sensor to determine transepidermal water loss, skin conductance, and skin hardness [20]. They designed a pen-type scheme utilizing a humidity sensor to collect and calculate the water evaporated from the skin. This regards the human skin as a resistor and applies current flows through the skin to obtain the resistance and conductance. They used a traditional force sensor and measured the force response of the prepared silicone blocks with different hardness values evaluated by the standard test. However, this device has a short measurement stroke, which can only measure the epidermis and not the depth of the fat and muscle layers. The method used to obtain hardness data was based on a comparison with standard data. The system was designed for a narrow and relatively hard area and was not suitable for evaluating soft tissues.

In our previous research [21], we developed a sensing system that can measure the self-defined stiffness of soft materials using a force visualization method. A silicone elastomer with a specific dimension was used as the benchmark material for the stiffness measurement. The sensing system used the darkness of the image to determine whether it was in contact. A camera was used to record the circular image data of the end face of the push rod. The relationship between the thrust and end-face area was measured in advance. In actual measurements, the pushing distance is indirectly obtained by calculating the area difference to calculate the stiffness from the start status to the contact status. However, the target softness was harder than the abdominal stiffness, and the issues of tissue flowing to the sides cannot be resolved by this sensor mechanism.

There are also other microdevices related to soft tissue property measurements. Fath El Bab et al. designed a micromachined piezoresistive tactile sensor for detecting the compliance of soft tissue, independent of the applied distance between the sensor and tissue [22]. Yang et al. presented a sensitive compliance measurement system for determining the stiffness of the adult rat hippocampus [23]. Peng et al. proposed a novel method of stiffness measurement by employing sensing elements with different stiffness values [24]. These sizes and ranges are different from our purpose, and the issues of tissue flow to the sides were not considered. An alternative approach is required.

Ultrasonic elastography (USE) and magnetic resonance elastography (MSE) have received significant attention in recent years for noninvasive assessment of tissue mechanical properties [25,26,27,28]. Niitsu et al. proposed a technique based on USE to produce a two-dimensional muscle hardness map [29]. These technologies convert the changes in the amplitude of the echo signal movement before and after compression into real-time color images based on the principle that different elastic coefficients between different tissues cause different deformations when compressed by external forces. However, the area of the abdominal muscle was too small to measure its stiffness. However, the effect of muscle stiffness on abdominal stiffness is limited. Therefore, USE and MSE cannot be used to assess the abdominal stiffness. Its high cost and low usability are also issues.

In view of the characteristics of the human abdomen and the specific research needs of abdominal stiffness evaluation in massage for slow-transit constipation, this study presents a new portable abdominal stiffness sensor suitable for evaluating abdominal stiffness, and reveals the relationship between the effectiveness of abdominal massage and change in abdominal stiffness by massage.

We designed a portable fixed-stroke abdominal stiffness sensor. It touches the participant’s abdominal skin through a force-measuring probe and a stopper. The probe generates the proper pressure through a preset spring to measure the depression deformation when the surrounding tissue makes contact with the stopper and calculates the stiffness of the abdomen. The probe has a simple and reliable structure and utilizes a low-cost small modular time-of-flight (ToF) sensor and high-precision spring elements to achieve deformation and force measurements. The stopper has contact detectors for evaluating the tissue stiffness as well as for achieving contact with a wide surface, preventing the target from flowing to the sides. The contact detectors are based on a capacitive skin contact judgment function, which enables accurate contact detection and measurement of the evaluation time. The sensor system also has a display screen to show the measurement results in real time, making the measurement process fast and easy. We used this sensor to measure the stiffness of key areas designated by the nurse before and after the nurse performed specific abdominal massages on the participants. Meanwhile, we tracked the defecation of participants before and after abdominal massage and analyzed the relationship between changes in abdominal stiffness and the effectiveness of abdominal massage.

## 2. Portable Fixed-Stroke Abdominal Stiffness (PFAS) Sensor

### 2.1. Functional Requirements 

The abdomen of the human body is poorly muscled and fatty, owing to the presence of many organs. Therefore, when the sensor probe is pushed into the abdomen, the tissue can flow to the sides. A mechanism is required to prevent these issues. The high softness of the abdomen should also be considered. To measure a large number of ordinary participants, it is desirable that the sensor system allow frequent daily measurements. To address these issues, the following functional requirements were defined:

1. Deformation and force are measurable
2. Be able to contact a wide surface, preventing tissue from flowing to the sides
3. Adequate for the abdominal softness of human body
4. Realize a simple, quick, comfortable, low-stress operation
5. Small in size, easy to carry, low cost, and easy to manufacture

### 2.2. Sensing Principle

**Figure 1:**
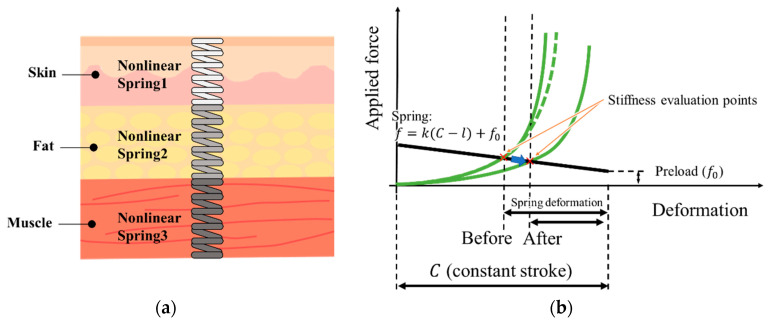
(**a**) Layers of abdomen. (**b**) Force–deformation curve for entire human abdominal wall with spring behavior indicating stiffness evaluation points.

The abdominal wall of the human body can be roughly divided into the epidermis, fat layer, and muscle layer, starting from the outermost layer, as shown in Figure 1. This can be equivalent to the model shown in Figure 1a, in which nonlinear spring1 corresponds to skin, nonlinear spring2 corresponds to fat, and nonlinear spring3 corresponds to muscle. According to the experimental process and requirements, we need to perform fast and effective comparative measurements of the hardness values of participants before and after receiving massage without subdividing each stiffness layer. Therefore, it is assumed that the abdominal wall measured in this study is regarded as a single skin–fat–muscle complex, and the sensor was developed based on this. To facilitate ease of understanding for people without sufficient technical background, including nurses, we defined abdominal stiffness in this study as the ratio of force acting on the abdominal surface to the depth of skin depression under the action of the force.

**Figure 2:**
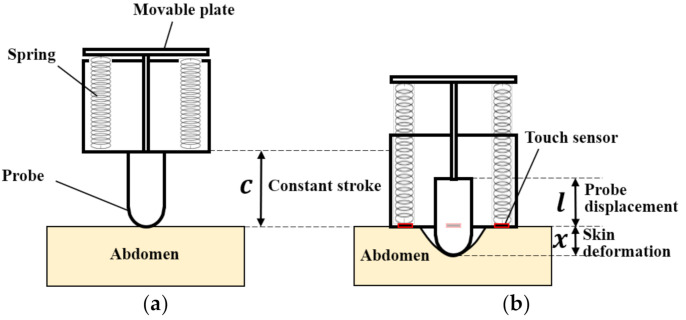
Schematic diagram of sensing principle: (**a**) Initial moment of measurement. (**b**) Final moment of measurement.

To obtain the stiffness, the force applied to the surface of the measured object and the corresponding depression deformation must be measured. The basic measurement principle is shown in Figure 2. A probe is used to push the piston rod’s linear feed mechanism and push the tension spring to generate a restoring force. The stroke for the probe was fixed from the moment when the sensor probe contacted the human abdomen to the end of the propulsion action, as shown in the equation

(1) C=x+l=const.,

where C is a constant stroke, x is the skin deformation caused by pressing, and l is the displacement of the probe that is pushed into the sensor. Different stiffnesses of the abdominal wall will produce different deformations, whereas the sum of the probe’s moving distance and the amount of skin deformation is always constant. Under the same spring, the pressing force F is a function of l ($F=f\left(l\right)$), and the corresponding stiffness is given by

(2) $$Stiffness=\frac{F}{x}=\frac{f\left(l\right)}{C-l},$$

where the relationship $F=f\left(l\right)$ is derived by the calibration described later. According to this principle, the points where the stiffness is evaluated by the sensor are represented by the black line shown in Figure 1b. According to conventional studies [30,31,32,33,34], the slope of the force–deformation curve for human skin–fat–muscle complexes, including abdominal ones, is small when the deformation is small. As the deformation increases, the slope increases rapidly (see green curves in Figure 1b). The intersection points of the black line and green curves represent the stiffness evaluation points, as shown in Figure 1b as red crosses. The case where the change occurs only after the sharp increase in the slope or the intersection of the black line and the green curve (see green dotted line in Figure 1b) cannot be evaluated using this stiffness evaluation method. According to an interview with nurses, they suggested that the change in stiffness can be detected with soft touch (even when the deformation or applied force is small). Therefore, the targeted change in stiffness was assumed to be the change occurring before the sharp increase in the slope. However, in order for this stiffness evaluation method to be valid, the probe stroke (C), spring constant (k), and preload value ($f_{0}$) need to be set appropriately such that the change can be seen.

To obtain stiffness based on this principle, we need to resolve the issue of tissue flowing to the sides when pressing the target abdomen, which is caused by high softness in the abdomen. If the target tissue/fat flows to the sides, then its stiffness cannot be included in the evaluation of stiffness. Additionally, excessive pressing can cause inaccurate evaluations. To minimize the flow effect, we designed a contact-detection device (red parts in Figure 2). The device contributes to obtaining a uniform wide contact with the tissues around the target, which indicates uniform deformation. Hence, it prevents the target tissue/fat from flowing to one side. Note that a contact is only made near the end of the measurement stroke. It also reduces the variance of the evaluated stiffness during the measurement and prompts the end time of the pressing, and can also help make it convenient to perform the automatic calculation.

### 2.3. Overview of Portable Fixed-Stroke Abdominal Stiffness (PFAS) Sensor

**Figure 3:**
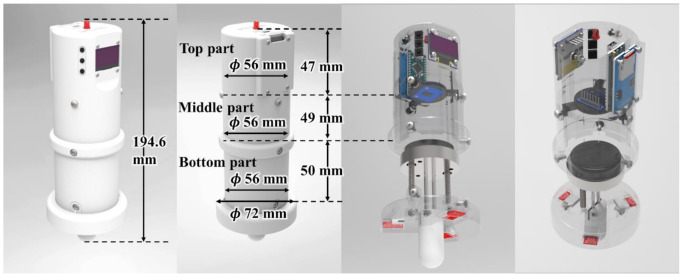
Appearance and internal structure rendering of portable fixed-stroke abdominal stiffness (PFAS) sensor.

The designed PFAS sensor is illustrated in Figure 3. The designed sensor can be divided into three functional parts: top, middle, and bottom. The fuselage was made via 3D printing (Raise 3D) using polylactic acid (PLA). A movable probe piston rod was installed at the bottom to measure stiffness. The contact detection device was located at the bottom of the bottom part. The operation panel, display, indicator light emitting diode (LED), and secure digital (SD) memory card reader were installed in the top area. Inside the sensor, there is a main control board, a ToF sensor, three touch sensors, an organic light emitting diode (OLED) display, several buttons, a metal piston rod, a metal fixed disk, and a probe that implements the sensing principle. The details of the sensor design are introduced next.

### 2.4. Sensor Design

**Figure 4:**
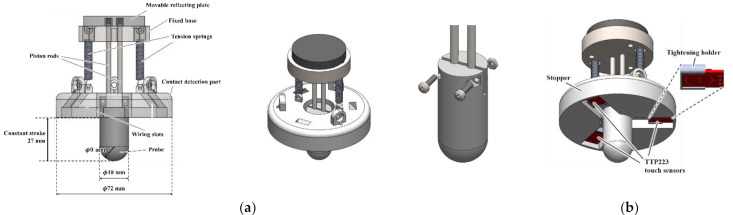
Structure of bottom parts: (**a**) Mechanical transmission structure of the probe. (**b**) Contact detecting part.

As shown in Figure 4, the mechanical transmission parts for the sensor probe and the contact detection system were mounted on the bottom part. The probe is used to directly contact the skin of the participant’s abdomen, generate a certain degree of small thrust force, cause the abdomen to sag within the elastic limit, and measure the depressed deformation. When the probe is pushed, the piston rod drives the movable reflecting plate, and two high-precision tension springs (E576, spring constant k=0.036 N/mm, pre-tension 0.441 N, Banecom, Japan) were then pulled to generate a thrust force for pressing into the abdomen and a restoring force. The moving distance of the movable reflecting plate is shown l in Figure 2b. Research [35] showed that the average fat thicknesses in the abdominal wall were 1.22 ± 0.55 cm and 1.94 ± 0.74 cm for men and women, respectively.

Considering that the abdominal tissue is mainly a fat layer, and most people suffering from constipation are female, the length of the probe or the fixed stroke distance was set to 27 mm, corresponding to the mean + 1σ: 19.4+7.4=26.8  mm. The spring constant and preload value (both specific values are given in Section 2.5) were selected such that the intersection between the force–deformation curve (green curve) and spring line (black line) in Figure 1b would be approximately half of this stroke length (27 mm) (refer to Section 2.5), so that the change in abdominal stiffness can be detected. The length of the stroke should be selected to be suitable for the target users (Japanese female in this study) so that a change in stiffness can be reliably detected. The probe was constructed using a cylindrical and hemispherical combination structure (Figure 4). The radius of the hemispherical part was 9 mm. The feed direction should be limited to a straight line. We designed two cylindrical piston rods made of duralumin to connect the probe and movable reflecting plate with a diameter of 38 mm. The two piston rods were inserted into two cylindrical through-holes (interference fit) in the middle of the fixed disc-shaped base made of duralumin to achieve linear movement. A lubricant (Silicone grease G501-80, Shinetsu, Japan) was used to obtain a smooth linear movement. To fix the probe and the push rod, we designed a bolt-and-nut locking structure that mimics the coupling type, which can be tightened repeatedly and disassembled multiple times to facilitate assembly, as shown in Figure 4a.

As mentioned above, a wide stopper with a diameter of 72 mm is needed to prevent tissue/fat from flowing out and obtain evenly distributed contact pressure at the skin around the target area. The stopper also contributes to the detection of skin contact. We designed a disc-shaped structure with a diameter larger than that of the main body as a stopper, as shown in Figure 4b. At the stopper, a contact detection system was installed to provide users with an intuitive contact detection signal to stop pressing and evaluate abdominal stiffness while pressing. The signal is also utilized as the input to the data recording system as a flag to start the valid data recording. The contact detection system was constructed using three capacitive detection contact sensors (TTP223, HiLetgo, Guangdong, China) embedded near the edge of the stopper. When all three sensors detect contacts, the three contact points constitute a wide contact surface around the target abdomen, which indicates a uniform deformation of the target abdominal area. Accordingly, the lateral outflow of the target tissue/fat can be minimized.

The detection timing also indicates the timing of stopping pressing to prevent excessive pressing. To inform the user of the timing, each contact sensor had a red LED light that lit up when the sensor detected a contact. Because the capacitance detection of the touch sensor is very sensitive, glue, metal screws, and other fixing methods will cause the capacitance to fluctuate. Therefore, a thin tightening holder was designed and printed with PLA, which is insensitive to capacitance. Preliminary tests demonstrated that the trigger distance of the touch sensor when it was stable was approximately 3–4 mm. Therefore, the depth of the touch sensor was set within the range of 3–4 mm from the lower surface, and fine adjustments were performed by adjusting the thin holder.

The middle part of the PFAS sensor has the following functions: measuring the moving distance of the movable reflecting plate, connecting the upper and lower parts of the sensor body, and facilitating the users to hold it.

**Figure 5:**
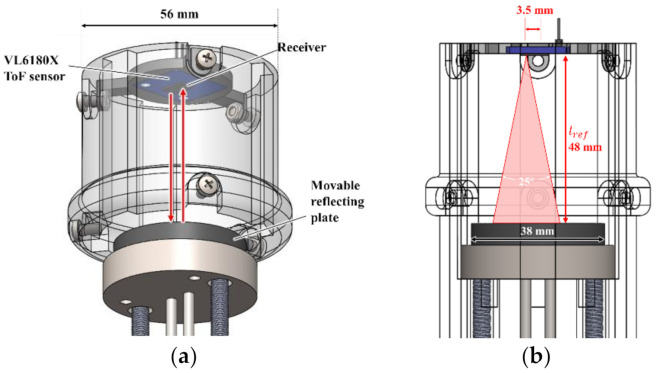
(**a**) Middle part for measuring moving distance of movable reflecting plate, connecting upper and lower parts of sensor body, and facilitating users to hold it. (**b**) Viewing cone of infrared receiver at ToF sensor (VL6180X).

As shown in Figure 5, the middle shell is 3D printed from PLA with a diameter of 56 mm, which is easy for users to hold. The moving distance of the movable reflecting plate, namely that of the probe, was detected using a time-of-flight (ToF) sensor (VL6180X, Adafruit, New York, USA) This sensor can emit modulated infrared light (IR), use the receiver to receive the reflected IR, and calculate the time of flight to achieve an accurate range (±1 mm), as shown by the red arrow in Figure 5a. Because the reliable working range of this sensor is 5–100 mm and the viewing cone of the infrared receiver is 25° (plane projection, as shown in Figure 5b), the distance of the ToF sensor from the reflecting plate $l_{ref}$ should be $5\;\mathrm{mm}<l_{ref}<100\;\mathrm{mm}$, and the maximum projection circle area at the farthest point should not exceed the top area of the reflecting plate. Therefore, we set the value of $l_{ref}$ to 48 mm.

As shown in Figure 5b, the distance of the infrared receiver from the circle center was approximately 3.5 mm, and the radius of the reflecting plate was set to 19 mm, considering $l_{ref}\;\times \;\tan12.5^{{}^{\circ}}\;+\;3.5\approx 14.141\;\mathrm{mm}<19\;\mathrm{mm},\;$ at which the distance measurement is safe and feasible. The PLA casing can be held by hand and protects the measurement space from being disturbed by external light. The surface of the reflecting plate was a natural PLA-printed texture. After extensive testing, the absolute distance data were found to be the most accurate when a black material was used. Accordingly, the movable reflecting plate was designed to have a black disc structure from the PLA. An exponential moving average (EMA) filter (smoothing factor, 0.8; time constant, 16 ms; sampling frequency, 38 Hz) was utilized to smooth the measured distance moved by the movable reflecting plate, and the moving distance of the probe corresponding to l in Figure 2b was derived. The EMA filter is given by

(3) $$l_{t}=\alpha \;l_{\mathrm{rt}}+\left(1-\alpha \right)l_{t-1},$$

where $l_{t}$ denotes the filtered l at time t, $l_{\mathrm{rt}}$ denotes the raw value of l at time t, and α denotes the smoothing factor.

**Figure 6:**
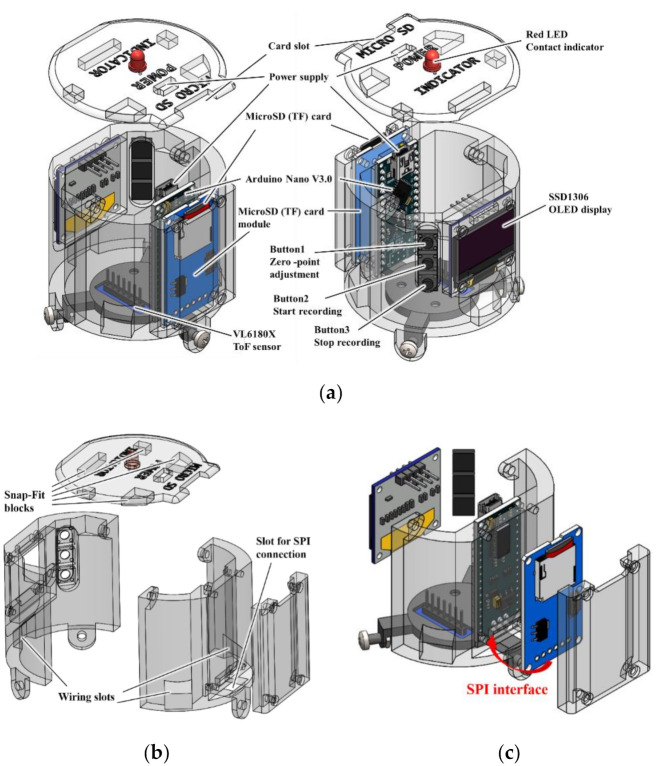
(**a**) Core control part and its composition structure. (**b**) Exploded figure of upper part. (**c**) SPI interface connection.

The top part of the PFAS sensor is the core control area. This part is mainly used for user operation, human–computer interaction, data transmission, storage, and control systems. The structural design needs to connect the distance measurement area in the middle part and realize the functions of fixing electronic components and parts. Given the cost considerations, we used the Arduino Nano for the main control board (Nano V3.0, HiLetgo). The remaining main components were an OLED display (SSD1306, Risym) and a Micro SD card module (HiLetgo). An SD card (32 GB) was inserted into the module. The power was supplied externally. Two lithium-ion batteries (NCR18650B 3400 mAh) were utilized by connecting it to the outlet for the power supply in the top panel. We designed the grooves and screw holes to fix the components, as shown in Figure 6a, to fix the components in accordance with the external dimensions of the components, keeping the premise that the location of the components did not affect the welding of the electronic circuit.

To facilitate assembly, the structure of this part is designed as a front-rear type, with a micro-SD-card-fixing part and a top cover. The four parts are spliced for easy assembly, disassembly, and maintenance. We designed three buttons set on the left side of the OLED display to correct the zero point of the sensor, start recording data, and stop recording data. The measurement is always active after power-on. Pressing the button 2 starts writing the measurement data to the SD card, and pressing the button 3 stops the writing. The display shows dynamic data in real time. Figure 6b shows the exploded views of each component. The entire three-stage casings of the sensor are designed with three wiring slots running through the sidewall of the entire sensor for wiring. A plug-in micro-SD card module can achieve serial peripheral interface (SPI) communication via a slot with the Nano. The fixation method of the top cover and casing was snap-fit, and a red LED was used as an indicator.

### 2.5. Force Calibration

**Figure 7:**
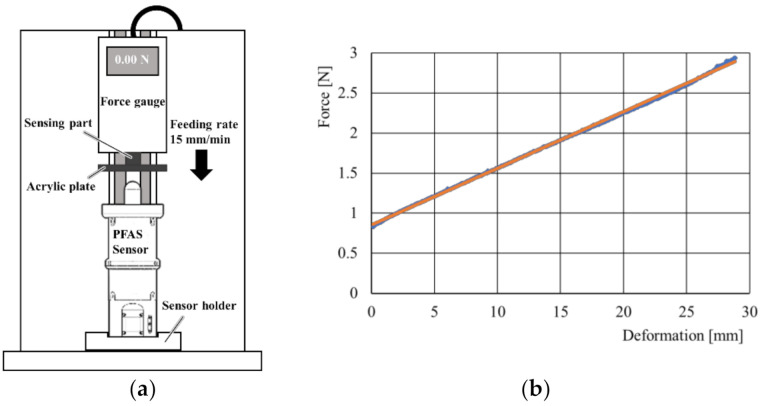
(**a**) Experimental setup for calibration test; (**b**) Force–deformation characteristic curve. The blue points represent raw data, whereas the red line represents the regression curve.

A calibration test was conducted to obtain the relationship $F=f\left(l\right),$ where F is the force applied by the probe, and l is the moving distance of the probe. The experimental setup is shown in Figure 7a. The force gauge was fixed on an experimental table equipped with a vertical slide rail. The vertical direction was controlled to move at a constant speed of 15 mm/min. The sensing part of the force gauge (IMADA ZTS-50N) was fixed to the acrylic plate so that the probe of the PFAS sensor could be pushed by the acrylic plate. The PFAS sensor was fixed to the bottom holder. The obtained data (blue points) can be approximated by a linear curve (red line), as shown in Figure 7b. The regression curve is shown in orange and is expressed as

(4) F=0.071l+0.85.

The obtained root mean squared error (RMSE) was 0.016 N, while the coefficient of determination was 0.995. The results demonstrate that the developed PFAS sensor has a high accuracy. The accuracy of the ToF sensor refers to the deviation of its value from the actual value. In the developed sensor, the measurement is performed inside the sensor; thus, the measurement target, measurement environment, and lighting conditions are always the same. Therefore, the deviation of the ToF sensor value from the actual value is the same in the measurement and can be included in the equation of the regression curve. Furthermore, the average value of the 15 filtered data was used for the calibration. Therefore, the accuracy of the developed sensor may be higher than that of the ToF sensor. Note that there are small differences in the manufacturer’s nominal values. The intercept of the obtained regression curve (0.85 N) was very close to the nominal values of the pre-tension (0.441× 2 = 0.882 N) of the spring. This result suggests that the effect of friction on the linear movement of the probe is very small owing to the lubricant.

The PFAS sensor was inverted during the test, and the sensor weight was compensated for. The total weight of the part that should be compensated was 29.77 g (0.292 N); thus, Equation (4) is corrected to

(5) F=0.071l+1.1.

From Equation (2), the abdominal stiffness defined in this study can be expressed as

(6) $$Stiffness=\frac{F}{x}=\frac{F}{C-l}=\frac{0.071l+1.1}{27-l}.$$

The constant stroke of the probe was set to C=27 mm.

**Figure 8:**
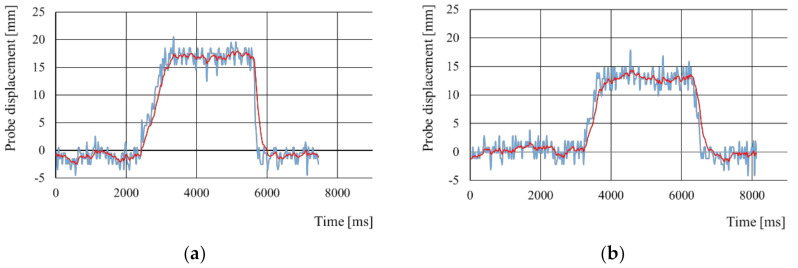
Example of probe displacement when measuring stiffness near left iliac bone of a participant: (**a**) before abdominal massage; (**b**) after abdominal massage. The blue line represents raw data, whereas the red line represents the filtered and smoothed data.

Figure 8 shows an example of probe displacement when measuring the stiffness near the left iliac bone of a participant. Figure 8a displays the probe displacement before the abdominal massage, whereas Figure 8b shows the displacement after the massage. The blue line represents raw data, whereas the red line represents the data filtered and smoothed by the EMA filter (3). Abdominal stiffness is derived from the filtered data and (6).

## 3. Abdominal Stiffness Evaluation in Massage for Constipation

Utilizing the developed PFAS sensor, abdominal stiffness was evaluated in massage for constipation, along with the relationship between the changes in abdominal stiffness and the effectiveness of abdominal massage.

### 3.1. Participants

We recruited 17 females claiming constipation (age 41.1 ± 18.5 years, weight = 52.5 ± 8.52 kg (514 ± 83.5 N), height = 157.1 ± 6.7 cm, and BMI = 21.2 ± 2.7). The evaluation procedure and purpose were approved by the Medical Ethics Committee of Kanazawa University (No. 60). Information on physical conditions, such as height and problems with constipation, was obtained via a questionnaire. Information on the Bristol Stool Scale (BSS) [36] for constipation before and after massage was obtained from another questionnaire. The purpose of the experiment and the procedure were explained to the participants, and their consent was obtained in the form of a consent transcript.

The inclusion criteria are as follows:

- Those who can provide written consent for participation in this study.
- Those of age 20 years or older at the time of obtaining consent
- Those who complain of constipation.
- The exclusion criteria are as follows:
- People with chronic diseases that may be affected by massage.
- Those who cannot understand the contents of the explanation and consent transcript.

### 3.2. Procedure

The evaluation steps were as follows:

- Step 1: Check the Bristol Stool Scale (BSS) just before receiving the massage; no defecation in one week prior to the massage was rated as no defecation.
- Step 2: Measurement of abdominal stiffness.
- Step 3: Abdominal massage for 30 min.
- Step 4: Measurement of abdominal stiffness.
- Step 5: Check the Bristol Stool Scale (BSS) immediately after receiving the massage; no defecation within a week after receiving the massage was rated as no defecation.

**Figure 9:**
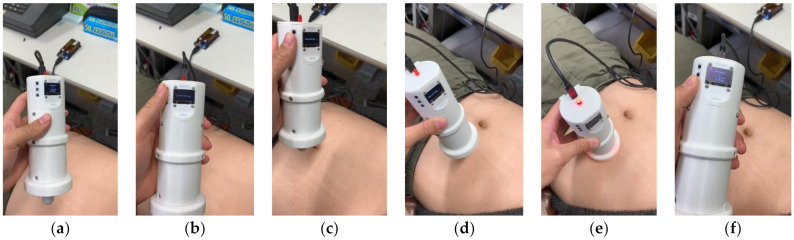
Evaluation steps for abdominal stiffness evaluation. (**a**) Power on and prepare to measure. (**b**) Correct zero-point value by pressing zero-point adjustment button. (**c**) Start recording by pressing record button. (**d**) Press specified area on abdomen. (**e**) Stop pressing when LEDs for all contact detection sensors are lit. (**f**) Stop evaluation and data recording by pressing stop button.

The developed PFAS sensor was utilized to measure the abdominal stiffness near the left iliac bone of the individuals, corresponding to the middle of the descending colon where the stool was formed. The reference point for the evaluation was the middle point of the descending colon between the left iliac bone and umbilicus. Before the measurement, the participant exposed her abdomen with the consent of the participant. The measurements were then conducted following the steps shown in Figure 9. We evaluated the area 10 times. Each measurement took approximately 10 s. During the measurement, we always observed the sensor contact detection situation, whether the participant was relaxed, whether the participant was talking or performing other actions, etc., to exclude invalid data. If the obtained data were invalid, then an additional measurement was conducted. Effects of viscosity can be avoided when the measurement starts some (short) time after end contact. After the viscous effects have settled, effects of hysteresis are avoided because the direction of the applied deformation in the measurement is always the direction in which the measured force increases.

Abdominal massage (see [37] for massage techniques and procedures) was conducted by a specialist (public health nurse) who was the coauthor of this study and provided constipation massage in home health care. Massage is usually performed until intestinal peristalsis is confirmed. For the evaluation, the duration of the massage was set to a constant 30 min, which was the average time to hear the sound of intestinal peristalsis in the preliminary experiments.

### 3.3. Data Analysis

We evaluated the abdominal stiffness of 17 participants. For the obtained 10 probe displacement data points for each (before and after the massage), the maximum and minimum values were removed, and the mean of the remaining data values was derived. From Equation (6) and the mean probe displacement, the abdominal stiffness is derived. To statistically observe the change in abdominal stiffness, a *t*-test for paired samples was performed. The significance level was set at p<0.05.

The Bristol Stool Scale (BSS) [36] was used to evaluate the effect of abdominal massage. The BSS is a widely used diagnostic tool that analyzes human stools to check whether a participant is constipated. The BSS was rated on a scale of 1 to 7. A BSS of 1 to 2 is considered constipation, 3 to 5 is normal defecation, and 6 to 7 is diarrhea. Based on this category, the effectiveness of the massage was evaluated in the format shown in Table 1. Among the 17 participants, 16 responded to the BSS questionnaire; thus, the analysis was conducted on the data for the 16 individuals who responded. To consider individual differences in comparing the change in abdominal stiffness with BSS, the change ratio of abdominal stiffness (CRAS) was utilized:

(7) $$\mathrm{CRAS}=\frac{\mathrm{Stiffness}\;\mathrm{after}\;\mathrm{massage}}{\mathrm{Stiffness}\;\mathrm{before}\;\mathrm{massage}}.$$

By comparing the value of CRAS with 1, the change in abdominal stiffness can be easily classified into three groups: decrease, maintenance, and increase.

### 3.4. Results

**Figure 10:**
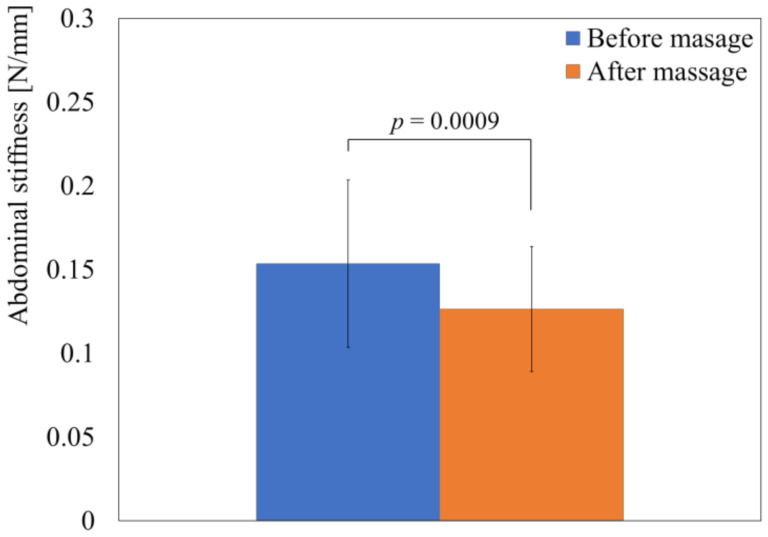
Evaluation results for abdominal stiffness. Error bar indicates standard deviation, and *p*-value shows results when performing *t*-test for paired samples.

First, the effect of massage on abdominal stiffness was evaluated. Figure 10 shows the results of the evaluation. Each bar represents the mean abdominal stiffness of the left abdominal areas corresponding to the middle area of the descending colon before and after massage. The error bars indicate the standard deviation of the obtained stiffness values. The obtained *p*-values are shown in Figure 10 (p=0.0009). The results indicate that massage significantly decreased the abdominal stiffness in the left abdominal area. The corresponding abdominal deformation at the stiffness evaluation points was 13±3 mm on average before the massage and 11±3 mm on average after the massage. Both values are approximately half of the stroke length (27 mm), and the significant difference in deformation (p=0.0006) obtained using a *t*-test for paired samples indicates that the change in abdominal stiffness due to the massage was detected by the developed sensor.

Second, we examined the relationship between the change in abdominal stiffness (CRAS: change ratio of abdominal stiffness) and changes in BSS. Unfortunately, the BSS for one participant was not available, and the examination was conducted for the remaining 16 participants. The results are presented in Table 2. CRAS <1 indicates decreased abdominal stiffness, CRAS  =1 indicates no change, and CRAS >1 indicates increased abdominal stiffness. ‘Remained in Good’ indicates that although the participants complained of constipation, their stool condition before and after the day they received the massage was healthy and there were no adverse effects of the massage. Hence, if abdominal stiffness was decreased by abdominal massage, then the stool condition improved, except for one exception (this case is discussed in the next subsection). In contrast, if there was no change in the abdominal stiffness owing to the massage, the stools remained in bad condition or worsened. One case of CRAS <1 is a possible exception and is discussed in the next subsection.

### 3.5. Discussion

In one case, when the BSS remained in a bad condition at CRAS <1, the BSS changed from 6 to 2. The questionnaire revealed that the participants complained of diarrhea rather than constipation. Additionally, the BSS was 2 immediately after the massage, but the stool conditions were reported to be healthy for several days after defecation, with a BSS of 2. Considering the change in stool properties, it is suggested that massage contributed significantly to the hardening of the stool, resulting in a poor condition. Considering the subsequent course of events, it can be said that this participant also improved in the stool condition. Namely, when abdominal stiffness was decreased owing to massage (CRAS <1), the stool condition was healthy or a healthy condition was maintained.

When abdominal stiffness was maintained irrespective of the massage (CRAS =1), the stool condition was maintained or worsened. For the evaluation, the duration of the massage was set to a constant of 30 min; thus, the duration was not sufficient for some of the participants, and more time was required to obtain the effect of the massage.

In one case, abdominal stiffness was increased by massage (CRAS >1). This participant had repeated diarrhea and constipation immediately before the massage; hard stool rated BSS 1, and diarrhea rated BSS 7. This complex gut environment may have influenced our results.

Participants were people of a wide age range, and their BMI ranges were normal. Therefore, the results are valid for a wide age range and women with normal body shapes. It can also be said that the developed stiffness sensor is valid in the abdomen of various thicknesses within the range of normal body shapes.

The effects of massage can be estimated by examining changes in abdominal stiffness, and thus, by observing the changes in abdominal stiffness, the method and duration of the massage can be changed appropriately during massage. However, it is unclear what changes in stiffness reflect. Relaxation of the rectus and transverse abdominis muscles from the umbilicus downward (lower abdomen) improves the circulation of blood flow, which results in increased intestinal peristalsis. Muscle relaxation is considered to be associated with reduced abdominal stiffness. Changes in abdominal stiffness could reflect changes in the fatty layer, peritoneum, stool properties, and stool volume. The details are beyond the scope of this study and may be involved in our future research.

## 4. Conclusions

This study presented an abdominal stiffness sensor to evaluate the benefits of abdominal massage immediately after massage for slow-transit constipation. The sensor consists of a probe, a spring, a stopper, and ToF sensors. The probe and spring were designed based on the statistics for the fat thickness of the abdominal wall so that appropriate pressure and deformation were applied to the abdomen, and the change in stiffness was evaluated. The stopper prevents the probe from being pushed into the abdomen more than necessary and includes contact detection systems to provide a wide area contact around the examination area to prevent the fat in the abdomen from flowing out to the sides.

The ToF sensor measures the deformation length from which the abdominal stiffness can be derived. With the developed sensor, this study also investigated the relationship between the change in abdominal stiffness and the effectiveness of abdominal massage. Although the abdominal feel of the constipated people softens after receiving abdominal massage according to the experience of nurses and physiotherapists, the relationship was not clear. The developed sensor was utilized to measure the abdominal stiffness near the left iliac bone of the individuals immediately before and after abdominal massage. The evaluated area corresponds to the middle of the descending colon, where the stool is formed.

The Bristol Stool Scale (BSS) before and after abdominal massage was used to evaluate the benefits of abdominal massage. The BSS is a widely used diagnostic tool that analyzes human stools to check whether a participant is constipated. The results indicated that a reduction in abdominal stiffness corresponds to an improvement in the stool condition or the maintenance of a healthy stool condition, whereas no change in abdominal stiffness indicates the maintenance or deterioration of the stool condition. The effects of massage can be estimated by examining changes in abdominal stiffness, and thus, by observing the changes in abdominal stiffness, the method and duration of the massage can be changed appropriately during massage. Further investigation and analysis are needed to determine the mechanisms by which abdominal massage affects abdominal stiffness, which may be relevant for our future research.

## Figures and Tables

**Table 1:** Method of comparison of changes in Bristol Stool Scale (BSS) before and after massage.

Evaluation Results	Before Massage	After Massage
Improved	BSS is 1 to 2 or BSS is 6 to 7	BSS is 3 to 5
	No defecation (one week)	There was a defecation
Remained in Good	BSS is 3 to 5	BSS is 3 to 5
Remained in Bad	BSS is 1 to 2 or BSS is 6 to 7	BSS is 1 to 2 or BSS is 6 to 7
Deteriorated	BSS is 3 to 5	BSS is 1 to 2 or BSS is 6 to 7

**Table 2:** Relationship between change in abdominal stiffness (CRAS: change ratio of abdominal stiffness) and change in BSS.

Change ratio of Abdominal Stiffness (CRAS)	Improved ^1^	Remained in Good ^2^	Remained in Bad ^3^	Deteriorated ^4^
CRAS<1 (Decreased)	4	7	1	0
CRAS=1 (Maintained)	0	1	1	1
CRAS>1 (Increased)	1	0	0	0

^1^ Stool condition was improved; ^2^ Stool remained in good condition; ^3^ Stool remained in bad condition; ^4^ Stool condition deteriorated.

## References

[1] Cabinet Office, Government of Japan Annual Report on the Aging Society: 2017 (Summary). https://www8.cao.go.jp/kourei/english/annualreport/2017/2017pdf_e.html.

[2] Bharucha A.E., Pemberton J.H., Locke G.R. (2013). American Gastroenterological Association Technical Review on Constipation. Gastroenterology.

[3] Rey E., Barcelo M., Jiménez Cebrián M.J., Alvarez-Sanchez A., Diaz-Rubio M., Rocha A.L. (2014). A Nation-Wide Study of Prevalence and Risk Factors for Fecal Impaction in Nursing Homes. PLoS ONE.

[4] Tanaka S., Yabunaka K., Matsumoto M., Tamai N., Noguchi H., Yoshida M., Nakagami G., Sugama J., Sanada H. (2018). Fecal Distribution Changes Using Colorectal Ultrasonography in Older People with Physical and Cognitive Impairment Living in Long-Term Care Facilities: A Longitudinal Observational Study. Healthcare.

[5] Research Society for the Diagnosis and Treatment of Chronic Constipation/Affiliated to the Japanese Society of Gastroenterology (2017). Evidenced-Based Clinical Practice Guidelines for Chronic Constipation.

[6] Read N.W., Celik A.F., Katsinelos P. (1995). Constipation and Incontinence in the Elderly. J. Clin. Gastroenterol..

[7] Digital Disimpaction and How It’s Done. https://www.verywellhealth.com/digital-evacuation-1945037.

[8] Silva C.A.G., Motta M.E.F.A. (2013). The use of abdominal muscle training, breathing exercises and abdominal massage to treat paediatric chronic functional constipation. Color. Dis..

[9] Lämås K., Lindholm L., Stenlund H., Engström B., Jacobsson C. (2009). Effects of abdominal massage in management of constipation—A randomized controlled trial. Int. J. Nurs. Stud..

[10] Wälchli C., Saltzwedel G., Krüerke D., Kaufmann C., Schnorr B., Rist L., Eberhard J., Decker M., Simões-Wüst A.P. (2014). Physiologic Effects of Rhythmical Massage: A Prospective Exploratory Cohort Study. J. Altern. Complement. Med..

[11] McClurg D., Hagen S., Hawkins S., Lowe-Strong A. (2011). Abdominal massage for the alleviation of constipation symptoms in people with multiple sclerosis: A randomized controlled feasibility study. Mult. Scler. J..

[12] Ernst E. (1999). Abdominal Massage Therapy for Chronic Constipation: A Systematic Review of Controlled Clinical Trials. Complement. Med. Res..

[13] Lämås K., Graneheim U.H., Jacobsson C. (2012). Experiences of abdominal massage for constipation. J. Clin. Nurs..

[14] Muller J., Ekström A., Harlén M., Lindmark U., Handlin L. (2016). Mechanical massage and mental training programs effect employees’ heart rate, blood pressure and fingertip temperature—An exploratory pilot study. Eur. J. Integr. Med..

[15] Viravud Y., Apichartvorakit A., Mutirangura P., Plakornkul V., Roongruangchai J., Vannabhum M., Laohapand T., Akarasereenont P. (2017). The anatomical study of the major signal points of the court-type Thai traditional massage on legs and their effects on blood flow and skin temperature. J. Integr. Med..

[16] Lee Y.-M. (2011). Effects of Self-Foot Reflexology on Stress, Fatigue, Skin Temperature and Immune Response in Female Undergraduate Students. J. Korean Acad. Nurs..

[17] Çevik K., Çetinkaya A., Yiğit Gökbel K., Menekşe B., Saza S., Tıkız C. (2018). The Effect of Abdominal Massage on Constipation in the Elderly Residing in Rest Homes. Gastroenterol. Nurs..

[18] TRY-ALL NEUTONE Muscle Hardness Tester TDM-N1/TDM-Z1(RB). http://www.try-all-jpn.com/english/hardness_meter/index.html.

[19] Sul O., Choi E., Lee S.-B. (2017). A Portable Stiffness Measurement System. Sensors.

[20] Sim D.J.K., Kim S.M., Kim S.S., Doh I. (2019). Portable Skin Analyzers with Simultaneous Measurements of Transepidermal Water Loss, Skin Conductance and Skin Hardness. Sensors.

[21] Watanabe T., Iwai T., Koyama T., Yoneyama T. (2016). Stiffness Measurement System Using Endoscopes with a Visualization Method. IEEE Sens. J..

[22] Fath El Bab A.M.R., Sugano K., Tsuchiya T., Tabata O., Eltaib M.E.H., Sallam M.M. (2012). Micromachined Tactile Sensor for Soft-Tissue Compliance Detection. J. Microelectromechanical Syst..

[23] Yang G.Y., Wen Y.-H., Foldy C., Tang W.C., Soltesz I. (2008). Sensor for Stiffness Measurements Within the Adult Rat Hippocampus. IEEE Sens. J..

[24] Peng P., Sezen A.S., Rajamani R., Erdman A.G. (2010). Novel MEMS stiffness sensor for force and elasticity measurements. Sensors Actuators A Phys..

[25] Yang Y., Qiu L., Wang L., Xiang X., Tang Y., Li H., Yan F. (2019). Quantitative Assessment of Skin Stiffness Using Ultrasound Shear Wave Elastography in Systemic Sclerosis. Ultrasound Med. Biol..

[26] MacDonald D., Wan A., McPhee M., Tucker K., Hug F. (2016). Reliability of Abdominal Muscle Stiffness Measured Using Elastography during Trunk Rehabilitation Exercises. Ultrasound Med. Biol..

[27] Gennisson J.-L., Baldeweck T., Tanter M., Catheline S., Fink M., Sandrin L., Cornillon C., Querleux B. (2004). Assessment of elastic parameters of human skin using dynamic elastography. IEEE Trans. Ultrason. Ferroelectr. Freq. Control.

[28] Venkatesh S.K., Ehman R.L. (2015). Magnetic resonance elastography of abdomen. Abdom. Imaging.

[29] Niitsu M., Michizaki A., Endo A., Takei H., Yanagisawa O. (2011). Muscle hardness measurement by using ultrasound elastography: A feasibility study. Acta Radiol..

[30] Fung Y.-C. (1993). Biomechanics.

[31] Deeken C.R., Lake S.P. (2017). Mechanical properties of the abdominal wall and biomaterials utilized for hernia repair. J. Mech. Behav. Biomed. Mater..

[32] Mezghani N., Husse S., Boivin K., Turcot K., Aissaoui R., Hagemeister N., De Guise J.A. (2008). Automatic classification of asymptomatic and osteoarthritis knee gait patterns using kinematic data features and the nearest neighbor classifier. IEEE Trans. Biomed. Eng..

[33] Förstemann T., Trzewik J., Holste J., Batke B., Konerding M.A., Wolloscheck T., Hartung C. (2011). Forces and deformations of the abdominal wall—A mechanical and geometrical approach to the linea alba. J. Biomech..

[34] Cardoso M.H.S. Experimental Study of the Human Anterolateral Abdominal Wall: Biomechanical Properties of Fascia and Muscles. https://repositorio-aberto.up.pt/bitstream/10216/65576/1/000154315.pdf.

[35] Mazziotti S., Cicero G., D’Angelo T., Marino M.A., Visalli C., Salamone I., Ascenti G., Blandino A. (2017). Imaging and Management of Incidental Renal Lesions. BioMed Res. Int..

[36] O’Donnell L.J., Virjee J., Heaton K.W. (1990). Detection of pseudodiarrhoea by simple clinical assessment of intestinal transit rate. BMJ.

[37] POOPOOLAND I’ll Teach You a Massage that Will Help You Clear Your Stool. https://www.youtube.com/watch?v=bsknMMGqfaw.

